# A temporal (phospho-)proteomic dataset of neurotrophic receptor tyrosine kinase signalling in neuroblastoma

**DOI:** 10.1038/s41597-024-03965-y

**Published:** 2024-10-10

**Authors:** Stephanie Maher, Kieran Wynne, Vadim Zhernovkov, Melinda Halasz

**Affiliations:** 1 Systems Biology Ireland, School of Medicine, University College Dublin, Dublin, Ireland; 2 Conway Institute of Biomolecular and Biomedical Research, University College Dublin, Dublin, Ireland

**Keywords:** Paediatric cancer, Proteomics, Cell signalling

## Abstract

Neurotrophic receptor tyrosine kinases (TrkA, TrkB, TrkC), despite their homology, contribute to the clinical heterogeneity of the childhood cancer neuroblastoma. TrkA expression is associated with low-stage disease and is often seen with spontaneous tumour regression. Conversely, TrkB is present in unfavourable neuroblastomas that often harbour amplification of the *MYCN* oncogene. The role of TrkC is less clearly defined, although some studies suggest its association with a favourable outcome. Understanding the differences in activity of Trk receptors that drive divergent clinical phenotypes as well as the influence of *MYCN* amplification on downstream Trk receptor signalling remains poorly understood. Here, we present a comprehensive label-free mass spectrometry-based total proteomics and phosphoproteomics dataset (432 raw files with FragPipe search outputs; available on PRIDE with accession number PXD054441) where we identified and quantified 4,907 proteins, 16,744 phosphosites and 5,084 phosphoproteins, derived from NGF/BDNF/NT-3 treated TrkA/B/C-overexpressing neuroblastoma cells with differential MYCN status. Analysing our dataset offers valuable insights into TrkA/B/C receptor signalling in neuroblastoma and its modulation by MYCN status; and holds potential for advancing therapeutic strategies in this challenging childhood cancer.

## Background & Summary

The neurotrophic receptor tyrosine kinases, also known as tropomyosin-related kinases or tropomyosin receptor kinases, are well characterised for their fundamental role during the development and maintenance of the nervous system^1,2^. These receptors, through their tightly regulated expression patterns and specific ligand-interactions, mediate critical processes such as neuronal cell differentiation, survival, and proliferation^1–3^. There are three homologous receptors; TrkA, TrkB and TrkC, encoded by the *NTRK1*, *NTRK2* and *NTRK3* genes, respectively. Each receptor demonstrates ligand specificity and binds with high affinity to distinct ligands known as neurotrophins. TrkA binds to nerve growth factor (NGF), TrkB to brain-derived neurotrophic factor (BDNF) and neurotrophin-4 (NT-4), and TrkC to neurotrophin-3 (NT-3). Upon ligand binding, classical receptor tyrosine kinase signalling ensues, including receptor homodimerisation leading to autophosphorylation in the tyrosine kinase domain and subsequent activation of the receptor. Adaptor proteins including Shc, Grb2 and SOS dock to the receptor facilitating the activation of downstream signalling cascades inclusive of Ras-MAPK, PI3K-Akt and PLCγ1^2^. When Trk receptor signalling goes awry, it can drive the development of various cancers, including neuroblastoma, where the receptors play distinct roles in tumour biology^4–7^.

Neuroblastoma is a rare but one of the deadliest paediatric cancers originating from neural crest cells of the developing sympathetic nervous system. Patients exhibit remarkable clinical heterogeneity, ranging from spontaneous regression of the tumour to aggressive metastatic disease^8,9^. Among others, expression of TrkA/B and amplification of the *MYCN* gene have been identified as important prognostic factors in neuroblastoma.

Despite structural and functional similarities, expression, and activation of Trk receptors result in divergent phenotypes of neuroblastoma cells and, in turn, influence clinical presentation. TrkA signalling induces differentiation of neuroblastoma cells^10^. Elevated expression of TrkA is associated with favourable neuroblastomas including low stage tumours, patients at lower age and lack of *MYCN* amplification^11–15^. Additionally, high TrkA expression is frequently present in neuroblastomas that spontaneously regress or differentiate into benign ganglioneuromas^16^. In contrast, expression and activation of TrkB with BDNF promotes an aggressive tumour cell phenotype characterised by enhanced cell growth, chemoresistance, invasion and angiogenesis^17–21^. TrkB is absent in low stage neuroblastomas and frequently present in *MYCN* amplified tumours^22,23^. The role of TrkC is not as well characterised in neuroblastoma. However, there is indication that TrkC expression is associated with low stage disease and a more favourable prognosis^4,24,25^. Hence, understanding the differences in Trk receptor signalling that leads to opposing cell fates (i.e., differentiation versus proliferation) and clinical features remains a long-standing challenge in neuroblastoma research.

Furthermore, the *MYCN* oncogene is central to the development of neuroblastoma. Amplification of the *MYCN* gene is present in 20–30% of all neuroblastomas and is the most significant independent prognostic factor of poor outcome^26,27^. Presence of *MYCN* amplification promotes an aggressive tumour environment characterised by metabolic plasticity, evasion of immune surveillance, stemness and resistance to cell death^28–31^. At the cellular level, MYCN has shown to exert profound reprogramming of the intracellular signalling networks and these differences were notably observed between *MYCN* non-amplified (including MYCN overexpression from single gene copy) and *MYCN* amplified neuroblastomas^32^. While previous studies have shown correlation between TrkA and TrkB expression and *MYCN* status in neuroblastoma^16,23^, the influence of *MYCN* status on the wiring of downstream Trk receptor signalling network is currently poorly understood. Here we provide a comprehensive phospho-(proteomics) dataset of each Trk receptor signalling network in different cellular contexts of neuroblastoma.

**Fig. 1:**
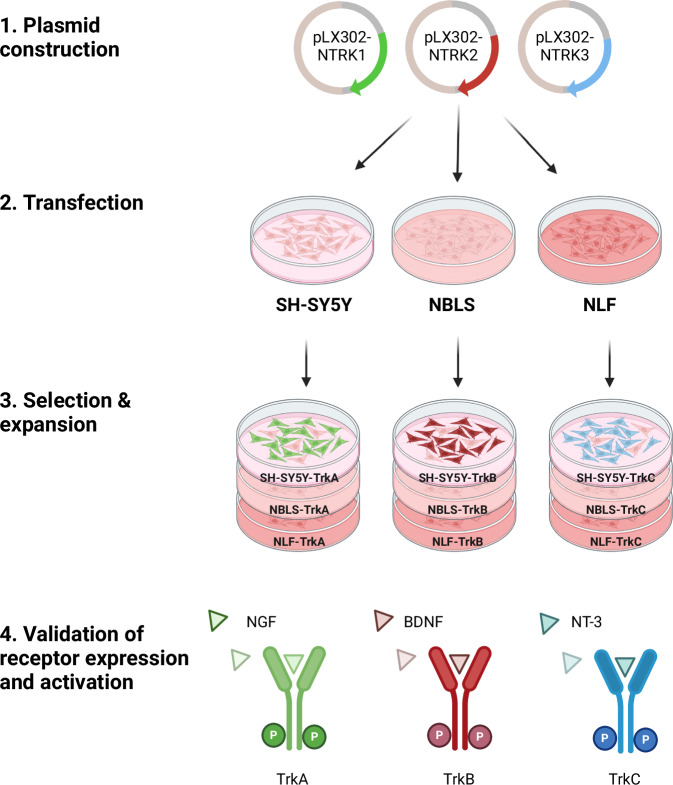
Schematic of the experimental steps preformed to establish and validate a Trk-expressing neuroblastoma cell system. Three cell lines with differential *MYCN* status (i.e., SH-SY5Y with single *MYCN* gene copy; NBLS with MYCN overexpression from single *MYCN* gene copy; and NLF with *MYCN* gene amplification) were used to generate nine cell lines that overexpress either the TrkA (gene: *NTRK1*), TrkB (gene: *NTRK2*) or TrkC (gene: *NTRK3*) receptor. TrkA, TrkB and TrkC receptor signalling were activated by NGF, BDNF or NT-3 ligand treatment, respectively. Figure created with BioRender.com.

First, we developed and validated an experimental cell system to study TrkA/B/C receptor signalling in neuroblastoma (Fig. 1)^10,33^. Neuroblastoma cell lines with different *MYCN* status and an absence of endogenous Trk expression were selected^22,34^. SH-SY5Y is a *MYCN* non-amplified neuroblastoma cell line. NBLS cells overexpress MYCN from a single gene copy, while NLF cells harbour *MYCN* amplification. Each of these cell lines were transfected with DNA constructs encoding the genes *NTRK1* (protein: TrkA), *NTRK2* (protein: TrkB) or *NTRK3* (protein: TrkC). Isogenic cell lines were subsequently established through antibiotic-based selection of positively transfected cells (Fig. 1).

**Fig. 2:**
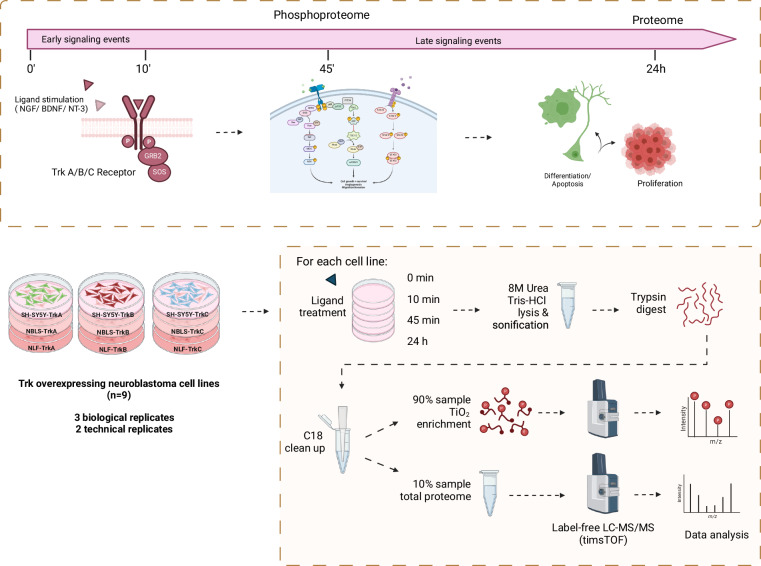
Overview of the total proteomics and phophoproteomics experimental workflow. (**a**) Time points of ligand stimulation used for temporal profiling of the TrkA/B/C proteome and phosphoproteome. Cells were serum starved (0.1% FBS) for 6 hours prior to ligand stimulation (100 ng/ml of NGF, BDNF or NT-3). (**b**) Workflow of mass spectrometry sample preparation and processing of matched proteome and phosphoproteome samples (9 cell lines, 4 time points, 3 biological replicates, 2 technical replicates resulting in 216 proteomics and 216 phophoproteomics raw files). Figure created with BioRender.com.

Following the successful development of a biologically relevant cell system, we investigated the temporal signalling dynamics and protein expression changes in these nine cell lines following ligand treatment (i.e., NGF for TrkA, BDNF for TrkB, NT-3 for TrkC) at four time points (0, 10 min, 45 min and 24 hours) using a combination of label-free mass spectrometry-based proteomics and phosphoproteomics approaches (Fig. 2). Here, we present the comprehensive dataset that consists of 2 × 216 LC-MS/MS runs, including three biological and two technical replicates of 36 conditions (i.e., 9 cell lines × 4 time points).

Phosphoproteomics allows for the unbiased identification and quantification of phosphorylated proteins, which are key players in signal transduction pathways. This approach offers insights into the activation and regulation of these pathways downstream of Trk receptor phosphorylation. Proteomics, on the other hand, provides an overview of the entire protein content within each Trk-expressing cell, capturing changes in protein abundance. Moreover, the specificity of cellular responses is often regulated by spatiotemporal dynamics of downstream pathway activity^35,36^. By stimulating the receptors with their ligands at early-, intermediate- and late-stage time points, this facilitates capturing the dynamic nature of the signalling events and uncovers an additional layer of potential cell fate regulation.

By combining these approaches, it is possible to delineate the complex signalling landscapes mediated by Trk receptor signalling in neuroblastoma and to understand how these pathways are influenced by different MYCN levels. For instance, we found that PKA signalling is crucial for inducing TrkC-mediated differentiation in non-*MYCN*-amplified NB cells; and showed that reactivation of the PKA pathway can induce differentiation of high-risk *MYCN*-amplified neuroblastoma^33^. We anticipate that this comprehensive dataset can be reused by researchers to enhance our understanding of Trk receptor biology in neuroblastoma and contribute to the development of targeted therapies for this challenging paediatric malignancy.

## Methods

### Cell culture

SH-SY5Y and NBLS parental human neuroblastoma cell lines were generously provided by Frank Westermann from Deutsches Krebsforschungszentrum (DKFZ), Heidelberg, DE, and Johannes Schulte (University Hospital Essen, Essen, DE). The NLF cell line was sourced from Kerafast (ECP008). Detailed characteristics of parental cell lines are available in Table 1. Parental cell lines were maintained in 5% CO_2_ at 37 °C and routinely cultured in RPMI 1640 (Gibco) supplemented with 10% (v/v) foetal bovine serum (Gibco), 2 mM L-glutamine (Gibco), and penicillin (100 U/ml) and streptomycin (100 μg/ml) (Gibco). Cell lines expressing the pLX302-NTRK1, pLX302-NTRK2 or pLX302-NTRK3 plasmid were additionally cultured with 1 μg/ml puromycin (Sigma-Aldrich) to ensure plasmid retention. Regular testing for mycoplasma was carried out on all cell lines.

**Table 1:** Characteristics of the parental human neuroblastoma cell lines used to generate the TrkA/B/C overexpressing cell lines.

Cell Line	Age of patient (in years)	Sex	Stage	*MYCN* Status	Met	Origin	Treatment
SH-SY5Y	4	Female	4	Single gene copy	Yes	Bone marrow	CT/RT
NBLS	3.6	Male	3	Overexpressed from single gene copy	No	Adrenal	None
NLF	3	Male	3	Amplified (HSR)	No	Abdomen	None

HSR = homogeneously staining regions; MET = metastasis; CT = chemotherapy; RT = radiotherapy.

### Construction of plasmids

The pLX302 destination vector (#25896) and entry clones pDONR223-NTRK1, pDONR223-NTRK2, pDONR223-NTRK3 (#23891, #23883, #23901, respectively) were obtained from Addgene, deposited by William Hahn & David Root^37^. The resulting expression clones (pLX302-NTRK1, pLX302-NTRK2, pLX302-NTRK3) were generated by the LR recombination reaction (Gateway® Cloning Enzyme mix, Life Science Technologies). Plasmid purification was carried out using PureYield Mini Prep (Promega) according to the manufacturer’s instructions.

### Generation of stable cell lines

Cells were seeded 24 h before transfection in 100 mm dish format and grown to 60–80% confluency. Cells were transfected with 5 μg of DNA using the jetPrime transfection reagent (Polyplus) following the manufacturer’s protocol. After 24 h, the media was replaced by RPMI 1640 complete media containing puromycin (1 μg/ml) to select for successfully transfected cells. The Trk-expressing cell lines were used between passage numbers 3–8 in the experiments.

### Ligand stimulation and cell lysis

For Western blot analysis, cells were seeded in 6-well dish format at a density of 1.5 × 10^5^ cells/ml and grown overnight to 80–90% confluence. For downstream phosphorylation analysis, cells were serum starved by replacing the cell culture media with media containing 0.1% FBS for 6 h prior to ligand treatment. After serum starvation, cells were treated with their respective ligands, i.e., NGF/BDNF/NT-3 (100 ng/ml) (Peprotech) for time-course stimulation. At time 0′, media was removed, cells were washed briefly with ice cold PBS and placed on ice. Cells were lysed in 110 µl ice cold lysis buffer (1% Triton x100, 20 mM Tris-HCl pH 7.5, 150 mM NaCl, 1 mM MgCl_2_), supplemented with protease inhibitor, cOmplete™ Mini (Roche) and phosphatase inhibitor, PhosStop™ (Roche). Lysates were centrifuged at 14,000 rpm at 4 °C for 10 min. The supernatants were then stored at −20 °C until further analysis.

### Western blot

Cell lysates (10 µg) were resolved by SDS-PAGE on 10% Bis-Tris Bolt gels (Invitrogen) connected to a BioRad power pac 300 at 110 V. Gels were transferred onto PVDF membranes (Millipore) at 30 V, 70 min using the XCell SureLock Electrophoresis Cell system (Invitrogen). Membranes were blocked in 5% non-fat dried milk (Millipore) for 1 h at room temperature prior to overnight incubation at 4 °C with primary antibody diluted in bovine serum albumin (BSA) (1:1000). Antibodies used include TrkA (#2505), TrkB (#4603), TrkC (C44H5) (#3376), phospho-TrkA^(Tyr490)^/TrkB^(Tyr516)^ (C35G9) (#4619), pERK^(Thr202/Tyr204)^ (#4370), pAkt^(Ser473)^ (#4060) and GAPDH (14C10) (#2118), all from Cell Signalling Technology. V5-tag monoclonal antibody (#R960-25) and PLCγ^(Tyr783)^ (#44-696) antibody were from Invitrogen. Secondary horseradish peroxidase–conjugated antibodies against rabbit (#7074) or mouse (#7076) immunoglobulin G (IgG) were from Cell Signalling Technology. Membranes were washed in TBS-Tween (3 × 5 min) then incubated with the corresponding secondary antibodies diluted in 5% milk (1:5000) for 1 h at room temperature. Blots were washed and then developed using the iBright CL750 Imaging System (Invitrogen) and 1:1 ratio of Pierce™ ECL Western Blotting Substrate or SuperSignal™ West Femto Maximum Sensitivity Substrate (Thermo Scientific). Quantification of blots was achieved using ImageJ software v1.44p (http://imagej.nih.gov/ij). Results were normalised to the loading control (GAPDH).

### Mass spectrometry sample preparation

Cells were seeded in 145 mm dishes for total proteomics and phosphoproteomics experiments. After 6 h of serum starvation (0.1% FBS), ligands - NGF/BDNF/NT-3 (100 ng/ml) (Peprotech #450-01, #450-02, #450-03) were added to the respective cells for time-course stimulation. At time point 0′, cells were detached with versene (500 ml PBS and 1 ml 0.5 M EDTA sterile filtered, pH 7.0) and centrifuged (300 g × 5 min, 4 °C). The supernatant was removed, and the cell pellets were resuspended in ice cold PBS. The pellet was centrifuged again (300 × g, 5 min, 4 °C) and the cell pellets were stored at −80 °C prior to sample preparation for mass spectrometry.

Cells were resuspended in ice cold 8 M urea/50 mM Tris-HCL pH 8.0, supplemented with phosphatase and protease inhibitors (Roche). Sample were sonicated (Syclon µUltrasonic 975 Homogenizer) for 2 × 9 sec, 15%. Sample protein concentrations were normalised to 500 µg using the Pierce Protein BCA assay (ThermoFisher Scientific). Samples were reduced by addition of 8 mM dithiothreitol (DTT) (Sigma-Aldrich) in a thermomixer at 1,000 rpm at 30 °C for 30 min; and subsequently carboxylated by addition of 20 mM iodoacetamide (Sigma-Aldrich) for 30 min in the dark (thermomixer 1,000 rpm, 30 °C). Urea concentration was brought down to 2 M by diluting samples in 50 mM Tris-HCL pH 8.0. Sequencing Grade Modified Trypsin (Promega) was resuspended in 50 mM Tris-HCL at a concentration of 0.5 µg/µl and added to each solution. The samples were digested overnight (1:100 enzyme to protein ratio) with gentle shaking (thermomixer 850 rpm, 37 °C). The digestion was terminated by addition of formic acid to 1% final concentration and cleaned up using C18 columns (HyperSep SpinTip P-20, BioBasic C18, Thermo Scientific). 10% of the sample was dried down for total proteome analysis. The remaining 90% of each sample was further subjected to phosphopeptide enrichment with TiO_2_ (Titansphere Phos-TiO BµLk 10 µm, (GL 986 Sciences Inc, Tokyo, Japan). Each sample was incubated with TiO_2_ beads (1 mg TiO/100 µg peptide) for 30 minutes by rotation in 80% acetonitrile, 6% trifluoroacetic acid, 5 mM monopotassium phosphate, 20 mg/ml 2,5- dihydroxybenzoic acid, this step was carried out twice. The beads were washed 5 times in 80% acetonitrile/ 1% trifluoroacetic acid, before elution of the phosphopeptides with 50% acetonitrile, 7% ammonium hydroxide. The two eluents from each sample were then pooled and dried down in CentriVap Concentrator (45 °C, 30 min).

### Liquid chromatography tandem mass spectrometry (LC-MS/MS)

Samples were run on a Bruker timsTof Pro mass spectrometer connected to a Evosep One liquid chromatography system. Tryptic peptides were resuspended in 0.1% formic acid and each sample was loaded on to an Evosep tip. The Evosep tips were placed in position on the Evosep One in a 96-tip box. The autosampler picks up each tip, elutes and separates the peptides using a set chromatography method (30 samples a day). The mass spectrometer was operated in positive ion mode with a capillary voltage of 1,500 V, dry gas flow of 3 l min^−1^ and a dry temperature of 180 °C. All data were acquired with the instrument operating in trapped ion mobility spectrometry (TIMS) mode. Trapped ions were selected for ms/ms using parallel accumulation serial fragmentation (PASEF). A scan range of (100–1,700 m/z) was performed at a rate of 5 PASEF MS/MS frames to 1 MS scan with a cycle time of 1.03 s^38^.

### Mass spectrometry data analysis

The mass spectrometer raw files were searched against the *Homo sapiens* subset of the Uniprot Swissprot database (reviewed) using the search engine FragPipe (18/19.1, latest version available at time of search)^39^.

### Bioinformatics

Data analysis was performed in R (Version 4.1.2). LFQ intensities were log2-transformed. Proteins/phosphosites with more that 80% missing values in all conditions were filtered out. Missing values were imputed using the group mean imputation with normal distribution correction and tail-based imputation approach. Analysis of differently expressed phosphosites and proteins was performed using the limma package in R/Bioconductor^40^ with adjusted p value < 0.05 and absolute fold change > 1.5/1 as the cutoffs for a phosphosite/protein to be considered significantly different compared to the zero timepoint of each experimental condition.

## Data Records

The mass spectrometry data has been deposited to the ProteomeXchange Consortium via the PRIDE^41^ partner repository with the dataset identifier PXD054441^42^. This dataset comprises raw files and processed files including tab-separated FragPipe output files as described in Tables S1, S2 (see supplementary xlsx files). Normalised and imputed data are available at figshare as outlined in Table 2.

**Table 2:** RStudio code and processed data deposited on figshare^43^.

File name	Description
experimental_design.rds	Experimental design of proteomics experiment
experimental_design_phospho.rds	Experimental design of phosphoproteomics experiment
Proteome_Preprocessing.R	Code for preprocessing the proteomics data
Phospho_preprocessing.R	Code for preprocessing the phosphoproteomics data
intensities.rds	Normalized and imputed proteomics data
phospho.rds	Normalized and imputed phosphoproteomics data
SciDataRMD.rmd	Code for processing of normalized data
SciData R script.R	Code for processing of normalized data

## Technical Validation

**Fig. 3:**
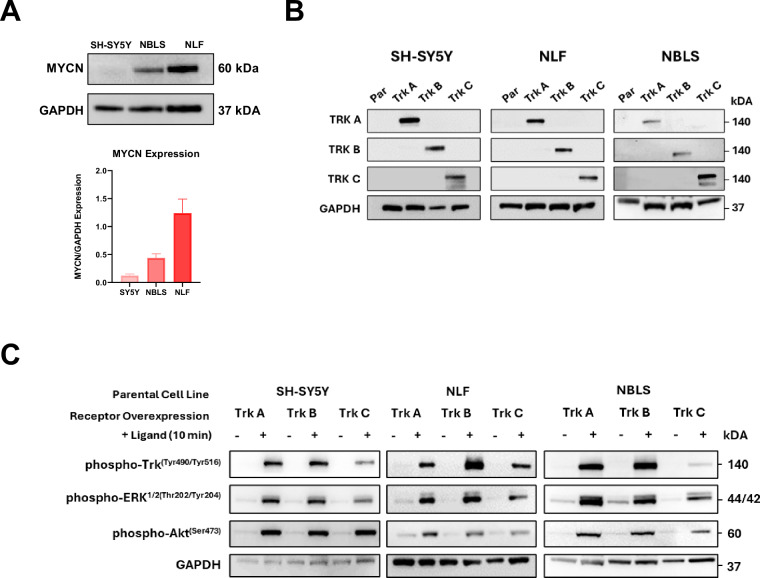
Validation of the TrkA/B/C-expressing neuroblastoma cell system. (**A**) MYCN protein expression across SH-SY5Y, NBLS and NLF, the parental cell lines used in the study, by Western blotting as shown by Maher *et al*.^33^ (**B**) Confirmation of TrkA/B/C receptor expression in each isogenic cell line by Western blotting. (Par: parental cell line) (**C**) Confirmation of Trk receptor activation following ligand stimulation (100 ng/ml; 10 min) of TrkA/B/C expressing cells and downstream activation of Akt and ERK by Western blotting. Cells were serum starved (0.1% FBS) for 6 hours prior to ligand stimulation. Ligands for TrkA, TrkB, and TrkC are NGF, BDNF, and NT-3, respectively. GAPDH acted as loading control for Western blotting.

The neuroblastoma cell lines SH-SY5Y, NBLS and NLF have different *MYCN* status, and MYCN protein expression levels were confirmed by Western blotting (Fig. 3A)^34^. The parental cell lines do not express either of the Trk receptors (Fig. 3B). We showed that the newly established SH-SY5Y/NTRK1, NBLS/NTRK1, NLF/NTRK1, SH-SY5Y/NTRK2, NBLS/NTRK2, NLF/NTRK2, SH-SY5Y/NTRK3, NBLS/NTRK3, NLF/NTRK3 cell lines overexpress the TrkA, TrkB or TrkC receptors (Fig. 3B). Additionally, Trk receptor functionality was demonstrated by the presence of Trk receptor phosphorylation following 10 minutes of ligand stimulation (NGF, BDNF or NT-3) and activation of downstream signalling pathways including phosphorylation of ERK, and AKT (Fig. 3C).

For the mass-spectrometry experiments, each isogenic cell line was exposed to their respective ligands for 0 minutes (unstimulated), 10 minutes, 45 minutes, and 24 hours to capture the dynamic range of downstream signalling events following receptor activation (Fig. 2a). To achieve comparative and reproducible-quality data, we utilised a matched proteome and phosphoproteome sampling strategy, where 90% of each sample was allocated for phosphopeptide enrichment using titanium dioxide (TiO2), and the remaining 10% was used for total proteome analysis. Additionally, samples were prepared in biological triplicates and run on the mass spectrometer in technical duplicates to assure for statistical power (Fig. 2b).

Label-free quantification was employed to assess changes in protein and phosphoprotein abundance across the different time points. Protein identification was conducted in data dependant acquisition (DDA) using the FragPipe computational platform with MSFragger. This approach led to the identification of 25,098 phosphosites and 5,084 phosphoproteins. Following data preprocessing and normalisation, we quantified 16,744 phosphosites (localisation probability > 0.75), providing a detailed and comprehensive mapping of phosphorylation events associated with TrkA/B/C receptor signalling over time (Table 3).

**Table 3:** Overview of phosphosite and protein identification & quantification.

Phosphoproteomics	Number of phosphosites identified	25, 098
	Phosphosites quantified after preprocessing	16, 744
	Phosphosite distribution by aa (%)	pS: 78.4%, pT: 18.0%, pY: 3.6%
	Phosphoproteins identified	5, 084
Proteomics	Proteins identified	6, 152
	Proteins quantified after preprocessing	4, 907

aa = amino acid; p = phospho; S = Serine; T = Threonine; Y = Tyrosine.

**Fig. 4:**
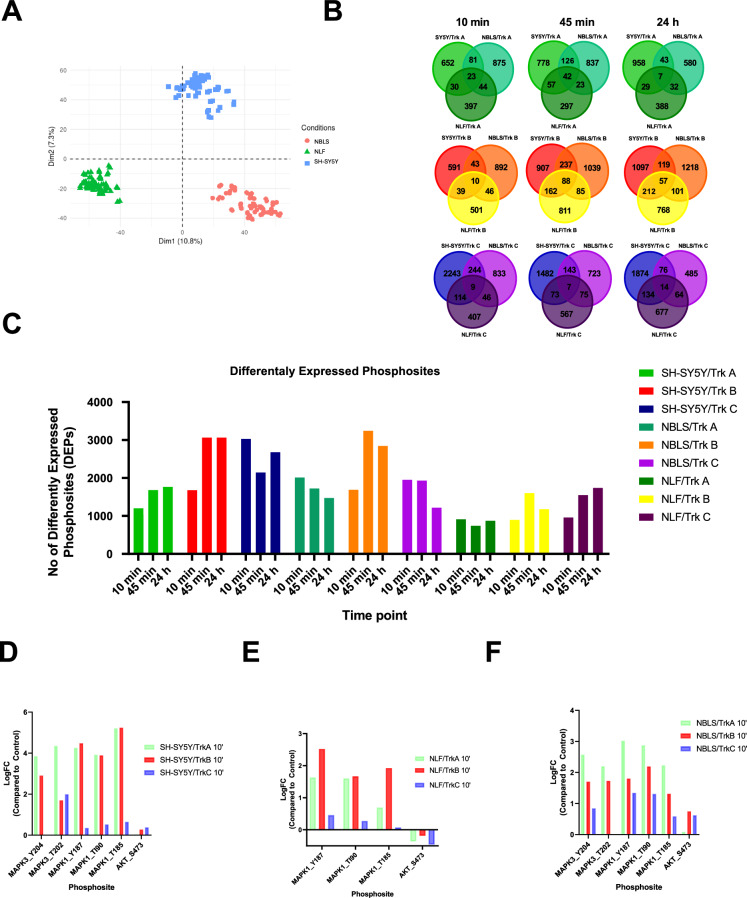
Overview of the phosphoproteomics data. (**A**) Principal component analysis of all samples (n = 216). (**B**) Overlapping upregulated differently expressed phosphosites (DEPs) (adjusted p-value < 0.05; absolute fold change > 1.5 from unstimulated cells) across cell lines at each time point of ligand stimulation. (**C**) Number of differently expressed phosphosites in each cell line at each time point of ligand stimulation. (**D**–**F**) Confirmation of LogFC of Akt and ERK1/2 phosphosites in each cell line compared to unstimulated control cells.

Principal component analysis indicates that samples cluster based on the parental cell line (Fig. 4A). Significant differently expressed phosphosites were calculated based on absolute fold change > 1.5 and adjusted p-value < 0.05 from unstimulated cells. Comparing differently expressed phosphosites across cell lines at each time point, there are both unique and overlapping phosphosites which have potential to be investigated further (Fig. 4B–C). Additionally, there is upregulation of phosphosites associated with putative downstream pathways following Trk receptor activation including pERK1/2^(Y202/204, T185/187)^ and pAkt^S473^ (Fig. 4D–F), confirming the processed phosphoproteomics dataset to accurately recapitulate Trk receptor signalling.

**Fig. 5:**
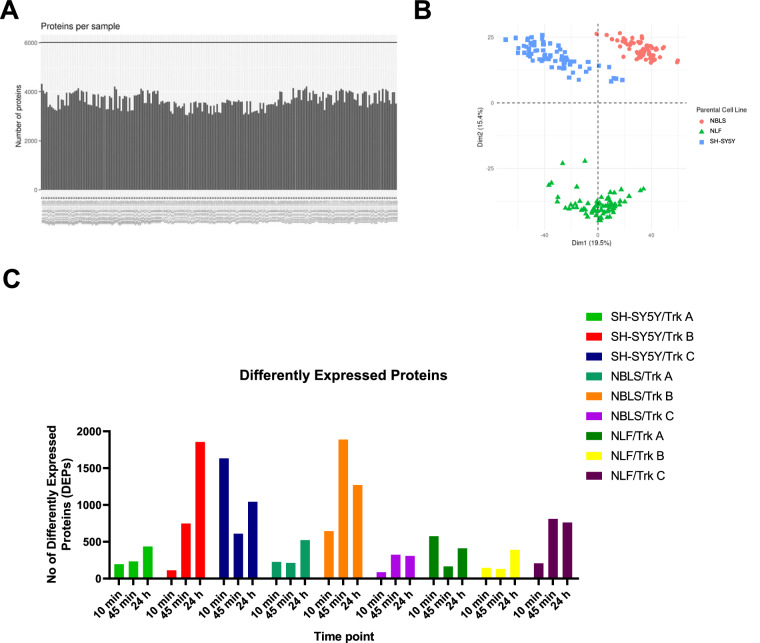
Overview of the total proteomics data. (**A**) Number of proteins identified in each sample (n = 215). To note, one of the technical replicates for the untreated NLF/NTRK1 total proteomics sample was removed after preprocessing and normalisation due to low number of proteins identified by MS. (**B**) Principal component analysis (PCA) of the samples (**C**) Number of differently expressed proteins in each sample at each time point (adjusted p-value < 0.05, absolute fold change > 1 from unstimulated cells).

The proteome data shows similar number of proteins identified across each sample (Fig. 5A). In correlation with the phosphoproteomic data, the proteome also showed separation based on parental cell line in the principal component analysis of all samples (Fig. 5B). The number of differently expressed proteins across each sample was also quantified (Fig. 5C) and showed differences between cell lines. MYCN amplified cells (NLF) showed an overall reduced amount of differently expressed proteins compared to SH-SY5Y and NBLS Trk expressing cells which may be in line with previous research showing MYCN to be a global suppressor of cellular signalling^32^.

In conclusion, this extensive dataset offers valuable insights into the temporal profiling and dynamic regulation of phosphorylation in Trk-mediated signalling in neuroblastoma. We envision that these datasets can be mined further to advance our mechanistic understanding of the Trk mediated signalling cues that control cell fate decisions in neuroblastoma and highlight vulnerabilities for therapeutic targeting.

## Usage Notes

One of the technical replicates for the untreated NLF/NTRK1 total proteomics sample (file name: *221012kw_SMaherProteomeNLF2_S1_A3_1_11597* in Table S2) was removed after preprocessing and normalisation due to low number of proteins identified by MS (n = 650). This is indicated by an asterix (*) in Table S1.

## Supplementary information

Supplementary Table S1

Supplementary Table S2

## Data Availability

RStudio code used to preprocess the mass spectrometry data is available in figshare^43^ (Table 2).
